# WiFi-Based Real-Time Calibration-Free Passive Human Motion Detection †

**DOI:** 10.3390/s151229896

**Published:** 2015-12-21

**Authors:** Liangyi Gong, Wu Yang, Dapeng Man, Guozhong Dong, Miao Yu, Jiguang Lv

**Affiliations:** The College of Computer Science and Technology, Harbin Engineering University, Harbin 150001, China; gongliangyi@hrbeu.edu.cn (L.G.); mandapeng@hrbeu.edu.cn (D.M.); dongguozhong@hrbeu.edu.cn (G.D.); yumiao@hrbeu.edu.cn (M.Y.); lvjiguang@hrbeu.edu.cn (J.L.)

**Keywords:** physical layer information, device-free passive, human motion detection

## Abstract

With the rapid development of WLAN technology, wireless device-free passive human detection becomes a newly-developing technique and holds more potential to worldwide and ubiquitous smart applications. Recently, indoor fine-grained device-free passive human motion detection based on the PHY layer information is rapidly developed. Previous wireless device-free passive human detection systems either rely on deploying specialized systems with dense transmitter-receiver links or elaborate off-line training process, which blocks rapid deployment and weakens system robustness. In the paper, we explore to research a novel fine-grained real-time calibration-free device-free passive human motion via physical layer information, which is independent of indoor scenarios and needs no prior-calibration and normal profile. We investigate sensitivities of amplitude and phase to human motion, and discover that phase feature is more sensitive to human motion, especially to slow human motion. Aiming at lightweight and robust device-free passive human motion detection, we develop two novel and practical schemes: short-term averaged variance ratio (SVR) and long-term averaged variance ratio (LVR). We realize system design with commercial WiFi devices and evaluate it in typical multipath-rich indoor scenarios. As demonstrated in the experiments, our approach can achieve a high detection rate and low false positive rate.

## 1. Introduction

Device-free passive human detection is a burgeoning technology to detect whether humans, without any electronic instrument, exist in the area of interests. It has an increasing demand and holds greater potentials to many security- and safety-critical applications including intruder detection, assets protection, elder nursing, *etc.*, where device attachment is inconvenient or even impossible. With rapid development of WLAN technique, it is potential to realize ubiquitous indoor wireless device-free passive human detection.

Inchoate indoor wireless passive human detection employed accessible received signal strength indicator (RSSI) from MAC layer. RSSI is a coarse-grained and low-resolution feature. In the indoor space, wireless signal further undergoes constructive or destructive multipath fading, leading to RSSI unreliability [1,2]. When a person blocks a pair of transmitter (TX) and receiver (RX), RSSI of a link may decrease, increase, or even remain unchanged [3]. Recently, numerous researchers explore utilization of WLAN PHY layer information to achieve indoor device-free passive human motion detection [4,5,6]. The channel state information (CSI) of multi-carrier signals delineates multipath components, and are sensitive to changes of direct, reflecting or scattering signals. Compared with RSSI, CSI is successful to greatly improve range and accuracy of passive human motion detection.

However, developed state-of-the-art systems are still weak in rapid deployment and robustness to changes of environment itself. They either rely on deploying specialized systems with dense transmitter-receiver links or elaborate off-line training processes. The former needs professional, intricate deployment and latter maintenance, which is not suitable for ubiquitous indoor scenarios. The latter realizes real-time detection based on clustering, pre-calibration or static pattern. Clustering and pre-calibration still involve time-consuming and labor-intensive training efforts. Lightweight passive human motion detection only relies on a normal profile, and detects human motion by comparing current signal pattern with static signal pattern. But once indoor tiny environmental change occurs, it is needed to recalibrate static pattern, which is suitable to unstable home or office space and poses a challenge to human motion detection. Aforementioned problems block the development of real-time passive human motion detection in ubiquitous indoor scenarios.

Based on the above motivation, in this paper, we mainly explore how to leverage PHY layer information to implement an advanced indoor fine-grained real-time passive human detection (FRID) that can be rapidly deployed, independent of indoor diverse scenarios and any calibration or re-calibration. Meanwhile, considering ordinary and common scenarios of building and home, we agree that a small number of pairs of transmitter and receiver, even a single link, are better choice for indoor narrow spaces. Hence, we will realize the lightweight real-time passive human detection based on a single link without any pre-calibration or a normal profile.

Next, we face the first challenge that *how to realize real-time passive human motion detection without any calibration or static pattern*. Once a pair of TX and RX is deployed, the function of detection is started up. Intuitively, detection scheme only relies on **real-time data flow**, and detects **difference between adjacent packets**. So, a plentiful experiments had be done to discover the effect of human motion on real-time signals. Unfortunately, we observe that amplitude difference of adjacent packets is not sensitive enough to human motion. Amplitude is also related with indoor scenarios and dropping off sharply with increasing of distance between human location and line of sight. In order to achieve accuracy detection, calibration is inevitable. Hence, the second challenge is *which feature that is sensitive enough to human motion can be extracted from adjacent packets*. Except for the amplitude feature, we note that **phase feature** is theoretically more sensitive to human motion, especially to slow human motion. However, because it is impossible to accurately measure and correct synchronization errors of WiFi device and commercial wireless NICs, the raw phase information behaves extremely randomly throughout the feasible field. In this paper, we acquire sanitized and usable phase information by utilizing a linear transformation on the raw CSI to eliminate the significant random noise. By plenty of experiments, the new phase information extracted from adjacent packets is demonstrated to be more efficient to sense human motion.

In order to realize scenario-independent passive human motion detection, we extract ratio of phases between adjacent packets as basic feature. If the environment is static, the ratio should be equal to 1. Otherwise, when a person moves in a monitoring area, the ratio is more or less than 1. To improve the system robustness, we apply the principle on a sequence of packets during adjacent time windows. In the system design, we develop two novel real-time human motion detection schemes based on coefficient of variation of phases: Short-term averaged variance ratio (SVR) and long-term averaged variance ratio (LVR). The two schemes is successful to eliminate the calibration cost and helpful to rapid deployment. 

Our main contributions are summarized as follows:

(1) We propose real-time passive human motion detection via PHY layer information without any calibration. We take advantage of physical-layer channel features, considering temporal stability and phase sensitivity to human motion. FRID is appropriate for rapid deployment, independent of indoor diverse scenarios and robust to environment changes.
(2) We implement indoor fine-grained real-time passive human motion detection system employing phase information of CSI from commodity WiFi device. We develop two novel real-time detecting schemes based on coefficient of variation of temporal phase. FRID can successfully combat the negative effects of indoor multipath and work on a single communication link. To the best of our knowledge, we are the first to only utilize the WLAN-based phase information to realize real-time passive human motion detection without calibration.
(3) Extensive evaluations of FRID are conducted in two typical indoor scenarios. The experiment results demonstrate that FRID can achieve satisfactory performance that outperforms RSSI-based system.

In the remainder of this paper, the related work can be reviewed in Section 2, and a preliminary about passive human motion detection is provided in Section 3. In Section 4, we detail the investigation about how to achieve real-time passive human motion detection without calibration, and put forward a effective phase-based feature in the Section 5. Then we detailed the developed two types of real-time passive human motion detection in the Section 6. Section 7 evaluates the performance of FRID in two scenarios. Section 8 concludes the paper.

## 2. Related Work

Since Youssef *et al*. [7] introduced the concept of Device-free Passive (DfP) localization, DfP had been extensively and deeply revolutionized. A device-free passive localization system refers to being able to detect, track and identify entities without carrying any related device. The detection component, as the fundamental process, is an essential primitive that is needed by a wide range of emerging applications. In early time, most of human motion detection systems exploited handy signature, RSSI. To overcome shortcomings of RSSI, some researchers are exploring to achieve indoor CSI-based DfP human motion detection.

**RSSI-based Detection.** RSSI can be handily extracted from ZigBee or WiFi devices. In reference [7], authors achieved passive human motion detection based on moving average and moving variance of WLAN-based RSSI. Next, as the performance of system [7] degrades in a real environment, the authors proposed a Maximum Likelihood Estimator (MLE) to enhance the performance of the DfP system in real environments [8]. RASID [9] analyzed RSSI features and adopted a non-parametric technique in different environments to further improve the performance of detection. In reference [10], authors researched different intrusion patterns and proposed a joint intrusion learning approach based on multiple intrusion indicators to enhance the performance of intrusion detection. In addition, anther well-know RSS-based DfP detection is the Radio Tomographic Imaging (RTI) [11]. Recently, various based-RTI DfP detection and localization are developed, including the vRTI [12], kRTI [13], dRTI [3]. Despite of its handy access, RSSI is coarse-grained and fails to capture the multipath effects [1] in indoor environments. Most systems only employ dense-deployed networks to detect human presence, which needs much labor and devices and raises the cost of applications. Besides, due to the interference of multipath effects, the performance of based-RSSI systems is poor for human slow motion and fast motion.

**CSI-based Detection.** Towards more lightweight and accurate systems, recent works dived into the PHY layer and exploited CSI for DfP detection. FIMD [14] realized passive human motion via clustering technique based on the eigenvalues of similarity matrix of CSIs. Then Z. Zhou [15] proposed an omnidirectional passive human detection system through utilizing PHY layer features to virtually tune the shape of monitoring coverage. J. Xiao *et al*. [6] presented a *Pilot* system leveraging temporal stability and frequency otherness of CSI and integrating an Anomaly Detection block to facilitate the device-free feature. In reference [5], authors proposed a behavior-free passive motion detection system that identifies different human behaviors. FCC [16] explored the relationship between the number of moving human and the variation of CSI and achieved the crowd counting based on the Grey Verhulst Model. PADS [4] extracted available phase information of WLAN CSIs and combined the amplitude with the phase to improve the accuracy and robustness of DfP human detection.

To summarize, RSSI’s fundamental drawback blocks the pervasive application of DfP systems in rich multi-path environments, e.g., indoor scenarios. On the contrary, our focus in this study is a WiFi-based finer-grained DFPL. However, similar works rely on either an elaborate training process or collecting lots of packets to cluster, both of which bring about reduced convenience and applicability of human motion detection. Different from prior works, FRID takes advantage of phase feature of CSIs and realizes a lightweight real-time passive human motion detection, which can accurately detect human motion with dynamic speed.

## 3. Preliminary

In typical indoor scenarios, wireless signal propagates to the receiver through multiple paths. Each multipath signal generates varying time delay, amplitude attenuation, and phase shift. Hence, the received baseband signal in such scenarios can be expressed as [17]:

(1) $$h\left(\tau \right)=\sum_{i=1}^{N}a_{i}e^{-j\theta_{i}}\delta \left(\tau -\tau_{i}\right)+n\left(\tau \right)$$

where $a_{i}$, $\theta_{i}$ and $\tau_{i}$ respectively represent the amplitude, phase and time delay of the $i^{th}$ path signal. *N* is the total number of paths. n(τ) is complex Gaussian zero-mean noise. We suppose that the coherent receiver is synchronized to the LOS signal ($\tau_{los}=0$) [18]. Hence, the received signal is written as:

(2) $$h\left(\tau \right)=a_{los}\delta \left(\tau \right)+\sum_{i=1}^{N-1}a_{nlos,i}e^{-j\theta_{i}}\delta \left(\tau -\tau_{i}\right)+n\left(\tau \right)$$

where $\theta_{i}=2\pi f\tau_{i}=2\pi f\Delta d/c$. *f*, Δd and *c* denote the signal frequency, the excess propagation distance of the non-line-of-sight (NLOS) path, and the speed of light, respectively. If we further suppose that the propagation medium is constant, the amplitudes of individual multipath signals are constant. Hence according to Equation (2), the received signal can be effected by the phase *θ*, related with the distance factor Δd and the frequency of carrier signal.

**Figure 1:**
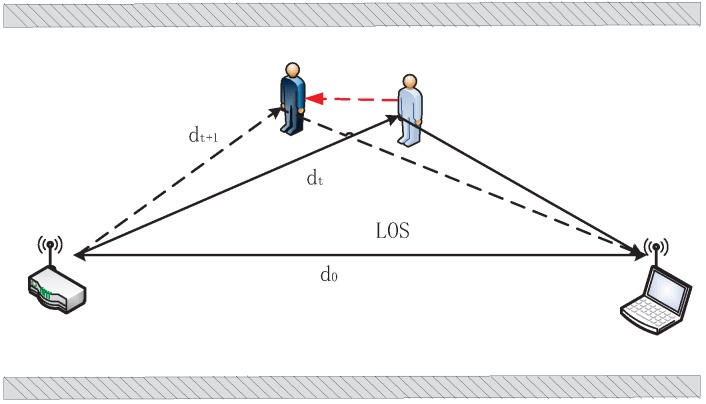
Single-bound reflection scenario.

Considering the most simplistic scenario, a single-bounce reflection caused by the human in addition to the Los signal, as depicted in Figure 1. If the frequency of carrier signal is constant, the phase is linear correlation with the factor Δd ($d_{t}-d_{0}$). When the human is walking, change of phase is continuous, leading to continuously variable received signal. The phase difference caused by human motion is denoted as $\Delta \theta =2\pi f(d_{t+1}-d_{t})/c$. That is when the path length of human moving changes by one wavelength every time, the receiver experiences a phase shift of 2π in the received subcarrier, which indicates that phase shift is more obvious and sensitive to human motion than amplitude change, especially to slow human motion.

The MAC layer RSSI serves as a prevalent indicator for channel quality, yet is a superposition of multipath signals and failed to capture the multipath effects. To detailedly depict the individual paths, a temporal filter, known as Channel Impulse Response (CIR) written as Equation (1), is employed to model the wireless propagation channel. However, commodity wireless infrastructure have not supported precise CIR estimation.

Fortunately, in the frequency domain, the channel model is characterized as Channel Frequency Response (CFR), which consists of amplitude-frequency response and phase-frequency response [15]. The *CSI Tool* based on the off-the-shelf Intel 5300 NIC and a modified firmware can successfully reveal a group of channel frequency response (CFR) [19]. Each CFR depicts the amplitude and phase of an OFDM subcarrier [20]:

(3) $$H\left(f_{k}\right)=∥H\left(f_{k}\right)∥e^{j∠H(f_{k})}$$

where $H(f_{k})$ is the CSI at the subcarrier with frequency of $f_{k}$, k∈[1,30]. $∥H(f_{k})∥$ and $∠H(f_{k})$ denotes the corresponding amplitude and phase of $H(f_{k})$ respectively.

Leveraging the off-the-shelf Intel 5300 NIC with a publicly available diver, the raw CFR divided into 30 groups can be expressed as:

(4) $$H\left(f\right)=\left[H_{f_{1}},H_{f_{2}},...,H_{f_{30}}\right]$$

where each $H_{f}$ is a complex number depicting the amplitude and phase of the subcarrier *f*. Simultaneously, although CIR and CFR are equivalent in modeling wireless channel, the CFR possesses more high sensitivity to human motion [15]. Hence, in this paper, we explore extraction of the CFR feature of WLAN signal for detecting the human motion.

## 4. Investigation

In order to explore real-time passive human motion detection without calibration or normal profile, we construct experiments in a complex indoor scenario. In the section, we detail the experiment set and observation.

### 4.1. Experiment Set

We constructed our experiment in a 12.4 m × 7.2 m classroom furnished with rows of desks and chairs. We employ a Tenda W3000R wireless router as a transmitter operating in IEEE 802.11n AP mode at 2.4 GHz. A laptop equipped with Intel 5300 NIC and 2 antennas running Ubuntu 10.04 LTS server OS works as a receiver pining packets from the transmitter. The transmitter and the receiver are placed 5 m away and 1.2 m in height. During our experiment, we set the transmission rate as 20 packets/s. The experiment was divided into two phases: Static phase and dynamic phase. During the static phase, no one was present in the classroom, and the receiver continuously collected 3000 packets in about 150 s. Then, when a volunteer slowly walked through direct link of TX-RX pair ten times, the receiver continuously collected 3000 packets in about 150 s.

### 4.2. Amplitude Variation

**Figure 2:**
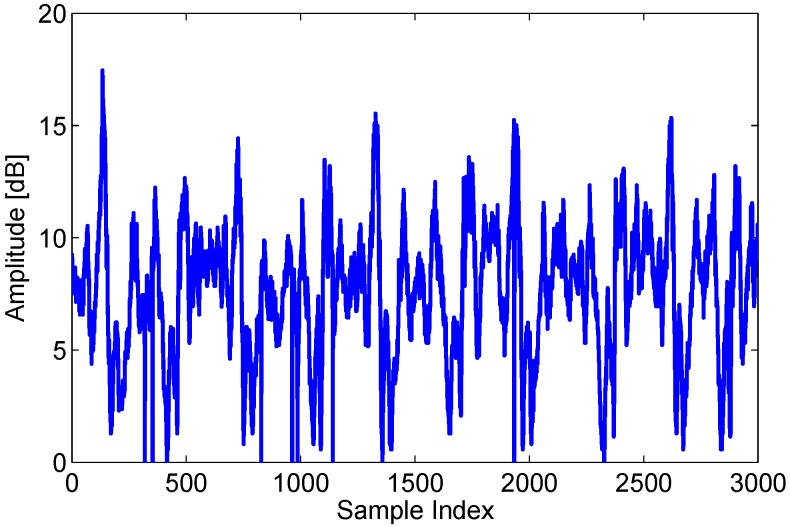
Amplitude variation of center frequency subcarrier in a dynamic state.

**Figure 3:**
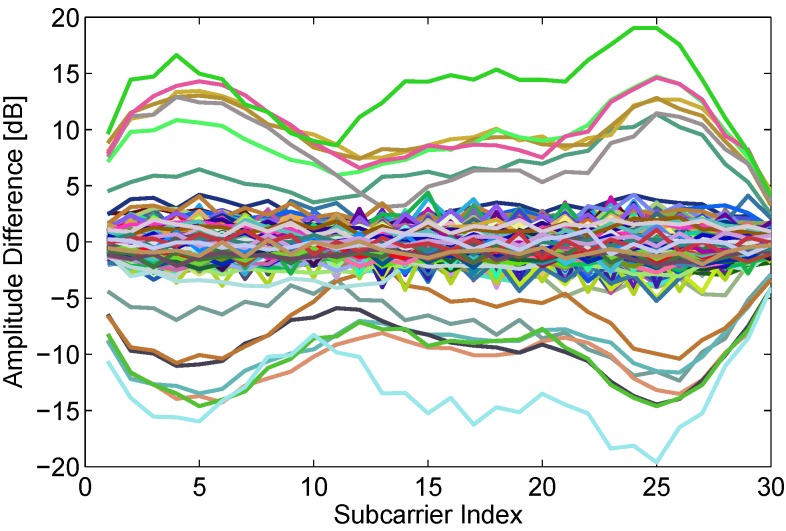
Amplitude differences between adjacent packets in the dynamic state.

Most of CSI-based device-free passive human motion detection systems pay more attention to the amplitude information due to the accessibility of amplitude information and the unavailability of phase information on commercial WiFi devices. So, we firstly investigate the effect of human motion on amplitudes of signals CSIs. The amplitudes of center frequency subcarrier are extracted from all packets of dynamic state and shown in Figure 2 where we can observe that human motion results in obvious interference to amplitude of CSIs. Aim at eliminating calibration and building normal profile process, we explore to employ the difference (as shown in Equation (5)) of CSIs between adjacent packets in real-time data flow to realize device-free passive human motion detection. The amplitude differences between adjacent packets in all packets of dynamic state are depicted in Figure 3. Unfortunately, we observe that only a dozen obvious difference curves exists but most of difference curves are extremely close to zero line, which is extremely different from that shown in Figure 2. The observation shows that amplitude difference feature extracted from real-time data flow is not sensitive enough to human motion. Hence, how to research another high-sensitive features from real-time data flow becomes a challenge.

(5) $$D_{i,j}=\left|\right|H_{i+1,j}\left|\right|-\left|\right|H_{i,j}\left|\right|$$

where *i* is index number of packet in real-time data flow, j∈[1,30] is index number of subcarriers.

### 4.3. Sensing Human Motion by Phase Feature

In this paper, we daringly explore to employ phase information of signals to realize real-time passive human motion detection. Due to the unavailability of phase information on commercial WiFi devices, we firstly solve a problem that how to extract available phase information. Then we will investigate the effect of human motion to phase features of signals.

#### 4.3.1. Phase Extraction

**Figure 4:**
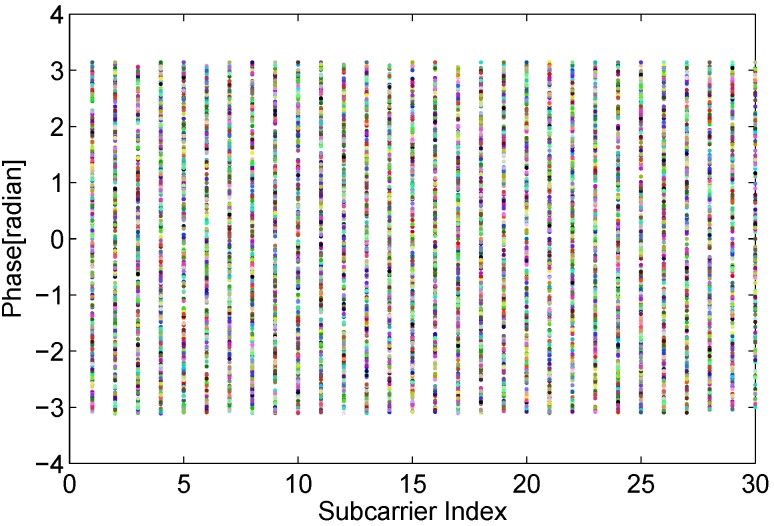
Random phase.

The measured phase $\tilde{\phi }$ of CFR for the $k^{th}$ subcarrier can be expressed as:

(6) $$\tilde{\phi_{k}}=\phi_{k}+2\pi \frac{k}{N}\tau_{\epsilon }+\lambda +n$$

where $\phi_{k}$ is real phase of $k^{th}$ subcarrier, *N* is the hits of FFT demodulator (which equals to 64 in IEEE 802.11 a/g/n), $\tau_{\epsilon }$ is the clock synchronization error that is proportional to the clock offset, *λ* is unknown constant phase error, *n* is some measurement noise. Because it is impossible to accurately measure and correct the synchronization error of a transmitter and a receiver, it is unfeasible to obtain true phase information with commodity WiFi NICs. The raw phase information behaves extremely random over the all feasible field as shown in Figure 4.

To obtain real phases of CFR, we employ a linear transformation on the raw phase to mitigate the random error ($\tau_{\epsilon },\lambda$), as recommended in [21]. As shown in Equation (6), the measured phase error $2\pi \frac{k}{N}\tau_{\epsilon }+\lambda$ is linear correlated with the subcarrier index *k*. To eliminate the *τ* and *λ*, we can utilize the slope *a* and intercept *b* of the linear phase [4]:

(7) $$a=\frac{\tilde{\phi_{k_{m}}}-\tilde{\phi_{k_{1}}}}{k_{m}-k_{1}}$$

(8) $$b=\frac{1}{m}\sum_{i=1}^{m}\tilde{\phi_{k_{i}}}$$

where *m* is the number of subcarriers. Due the slope *a* and intercept *b* respectively containing the real slope and intercept of phase, the processed phase $\hat{\phi_{k_{i}}}$ can be achieved when the measured phase subtracts the estimated linear error ($ak_{i}+b$):

(9) $$\begin{array}{cc} \hat{\phi_{k_{i}}} & =\tilde{\phi_{k_{i}}}-\left(ak_{i}+b\right)\\ & =\phi_{k_{i}}-\frac{k_{i}}{k_{i}-k_{1}}\left(\phi_{k_{n}}-\phi_{k_{1}}\right)-\frac{1}{m}\sum_{j=1}^{m}\phi_{k_{j}}\\ & -\frac{2\pi \tau_{\epsilon }}{mN}\sum_{j=1}^{m}k_{j} \end{array}$$

Supposing that the subcarriers index is symmetric, which indicates $\sum_{j=1}^{m}k_{j}=0$, the error term $\tau_{\epsilon }$ can be further removed. Hence, the final processed phase can be written as:

(10) $$\begin{array}{c} \hat{\phi_{k_{i}}}=\phi_{k_{i}}-\frac{k_{i}}{k_{i}-k_{1}}\left(\phi_{k_{n}}-\phi_{k_{1}}\right)-\frac{1}{m}\sum_{j=1}^{m}\phi_{k_{j}} \end{array}$$

**Figure 5:**
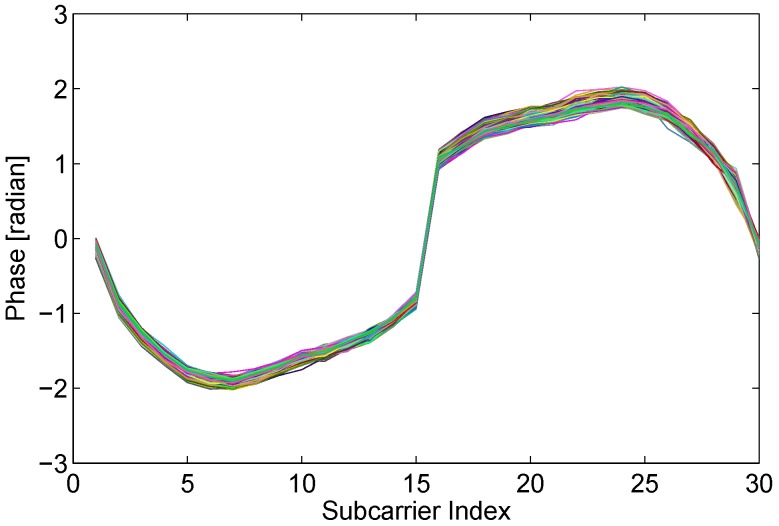
Phase before and after linear transformation.

Although we can not gain the fully true phase, we do derive a stable and available feature of phase information, as shown in Figure 5. Afterwards, we investigate whether the transformed phase is stable and sensitive enough to human motion.

#### 4.3.2. Phase Variation

**Figure 6:**
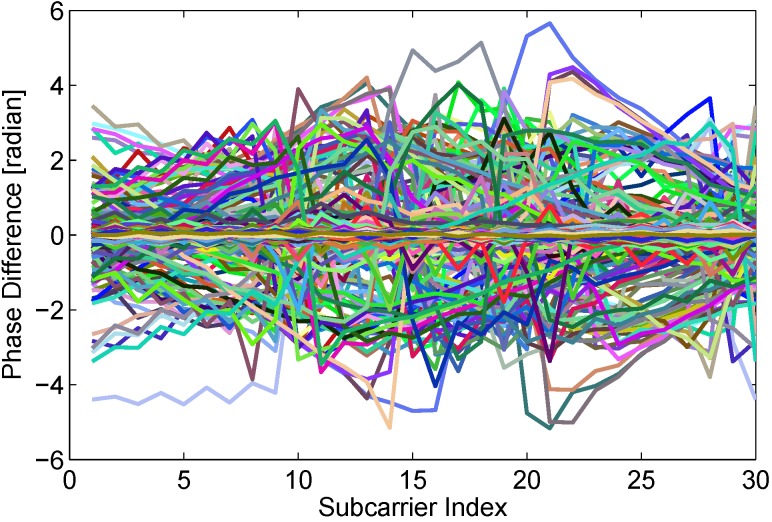
Phase differences between adjacent packets in the dynamic state.

To investigate phase variation caused by human motion, we extract available phase features from all packets in the dynamic state employing the approach introduced in above section. Then we calculate phase differences between adjacent packets, similar to the above-mentioned calculation process of amplitude differences. The phase differences of all subcarriers are plotted in Figure 6. Comparing Figure 6 with Figure 3, we can find that phase difference between adjacent packets is more sensitive to human motion than amplitude difference. The time interval between adjacent packets is very small, so the distance of human movement is very short. As described in the Section 3, human slight motion also brings obvious phase changes. However, amplitude of CSI is related with total power of multipath signals. When a person moves a short distance, the number of multipath signals changes slightly, which may not bring large power change.

**Figure 7:**
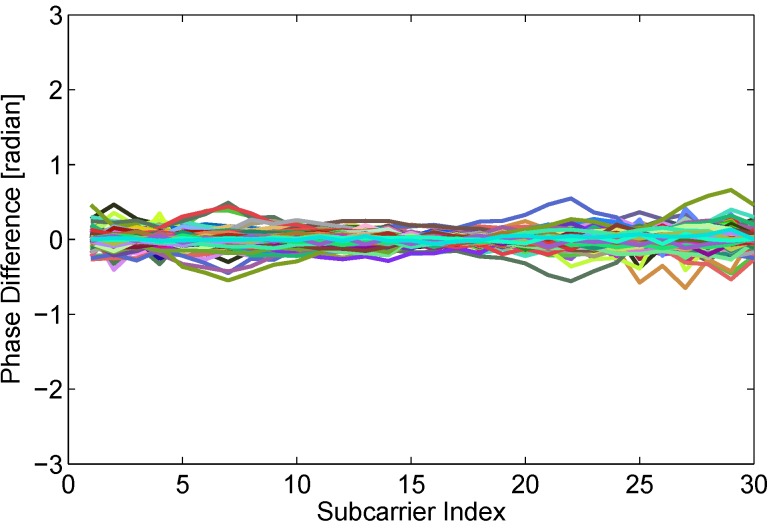
Stable phase difference in the static state.

As widely known, signals in a static state may be effected by environment noise and surrounding electromagnetic. The signal features jitter slightly, which is common and accepted for abnormal detection. However, a very unstable feature in a static state causes higher false positive, which is also unsuitable to real-time detection. To investigate the usability of phase difference feature between adjacent packets, we calculate phase differences of 30 subcarriers in 3000 packets of static state. As shown in Figure 7, almost all of phase difference curves are close to zero line.

Through aforementioned experimental investigations, we can see that phase difference between adjacent packets from real-time data flow is not only stable in a static state, but also sensitive enough to human motion. Hence, in the paper, we are going to give full play to the advantage of phase information to achieve real-time device-free passive human motion detection.

## 5. Feature Extraction

An appropriate feature is key for high performance of device-free passive human motion detection. In the section, we explore how to extract a detecting feature that is robust and sensitive to human motion. As discussed in above section, we can see that phase difference between adjacent packets is sensitive enough to human motion but not robust enough. Hence, in order to eliminate environment noise and improve system robustness, we employ a sliding time window scheme, and human motion can be detected by mean phase difference between adjacent time windows.

**Figure 8:**
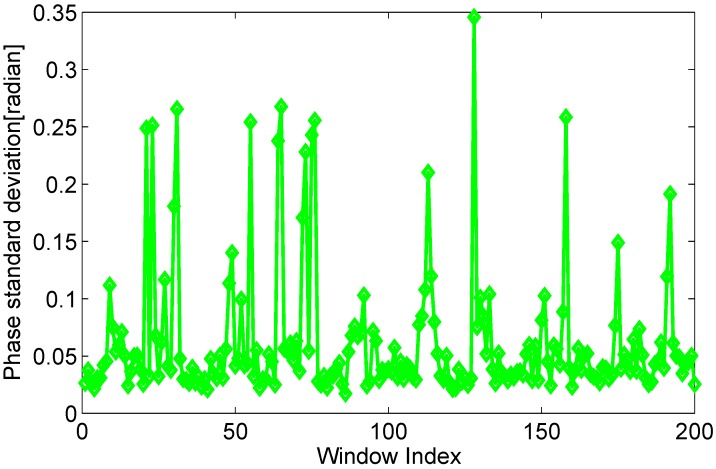
Phase variance of time windows in the dynamic state.

On the other hand, as depicted in Figure 1, when the human is moving, the Δd changes continuously. The phase in current time *T* is different from that in previous time $T^{{}^{\prime }}$, that is $\Delta d_{T}\neq \Delta d_{T^{{}^{\prime }}}$. The values of Δd in a dynamic state are discrete and fluctuant in a time window. Intuitively, the phase variance in a time window with high human motion speed is more violent than that with low human motion speed. The experimental observation shows that the phase variance in a time window also reflects human motion, as shown in Figure 8.

**Figure 9:**
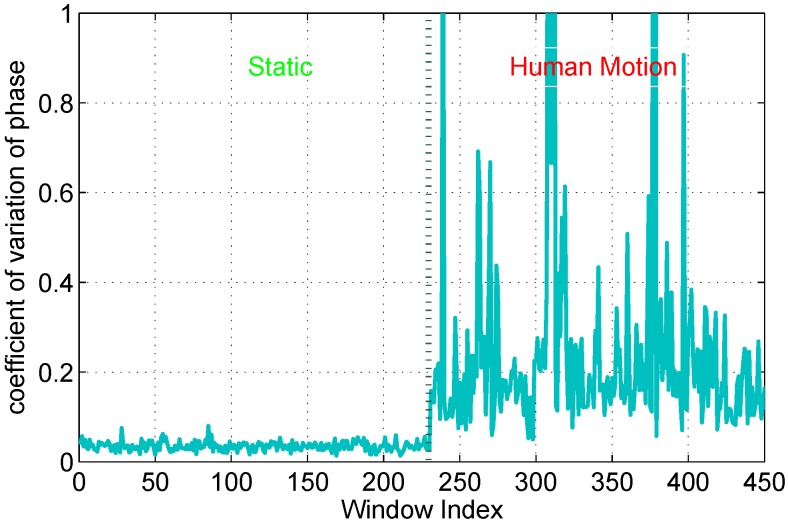
Coefficient of variation of phase in static/dynamic environments.

In the paper, we explore to utilize coefficient of variation (c.v.)of phase, which is reported as a percentage and calculated from the average and standard deviation of phase in a time window. The coefficient of variation of phase of the $k^{th}$ subcarrier is defined as:

(11) $$\delta_{c.v}^{k}=\frac{\sigma_{\Delta_{T}}^{k}}{\mu_{\Delta_{T}}^{k}}$$

where $\Delta_{T}$ is a sampling time window, $\sigma_{\Delta_{T}}^{k}$ and $\mu_{\Delta_{T}}^{k}$ are the standard deviation and mean of phase of the $k^{th}$ subcarrier in a time window $\Delta_{T}$. The coefficient of variation of phase can not only depict temporal discreteness of phase, but also eliminate the dissimilitude of measurement scale in different time windows, as shown in the Figure 9. Meanwhile, the new phase information is more stable, because of the linear transformation eliminating lots of random noise in CFRs. As depicted in Figure 9, the coefficient of variation of phase in a time window is more obvious to distinguish a static environment with a dynamic environment. Then we will develop real-time human motion detection scheme based on coefficient of variation of phase, which is detailed in the following section.

## 6. Real-Time Human Motion Detection

The performance of calibration-based methods such as site-survey, pre-calibration, constructing normal profile, *etc.*, can be greatly effected by changes of environment itself and locations of furniture, which are unsuitable for complex and changeable scenarios. And for that reason, in the paper, we try to realize real-time device-free passive human motion detection without any calibration.

We take advantage of coefficient of variation for detecting abrupt changes in the CSI phase for a WiFi link. Let $\delta_{\Delta_{T}}^{i,j}$ be the coefficient of variation for the *j*th transmitter-receiver antenna pair and the *i*th OFDM subcarrier for the $\Delta_{T}$ time window. In order to detect temporal coefficient of variation changes, we track $\delta_{\Delta_{T}}^{i,j}$ over a short-term window $\Delta_{T}$ and a long-time time window $\Delta_{LT}$, and define two types of schemes: short-term averaged variance ratio (SVR) and long-term averaged variance ratio (LVR), allowing us to compare the different temporal statistics of the WiFi link. The short-term window can reflect current state and be helpful to detect abrupt abnormal event [22]. The long-term window represents a stable state, and makes FRID void the re-calibration. Temporal variance ratio is a relative metric that is not affected by various environment conditions.

**Figure 10:**
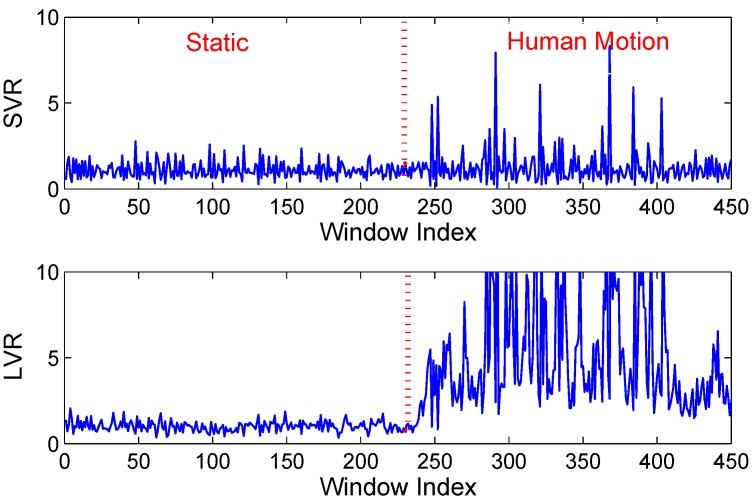
Variance of phase in static/dynamic environments.

We define short-term averaged variance ratio (SVR) of phase as:

(12) $$R_{SVR}=\frac{1}{n}\sum_{i=1}^{n}\left|\frac{\delta_{\Delta_{T}}^{i}}{\delta_{\Delta_{T-1}}^{i}}\right|$$

where *n* is the number of subcarriers, $\delta_{\Delta_{T}}^{i}$ and $\delta_{\Delta_{T-1}}^{i}$ are respectively the coefficient of variation of phase of $i^{th}$ subcarrier in time intervals $\Delta_{T}$ and $\Delta_{T-1}$. When the environment is static, the mean value of $R_{SVR}$ should be equal to 1. Due to inevitable noise from environment and hardware, values of measured $R_{SVR}$ fits a Gaussian distribution $R_{SVR}∼N\left(1,\sigma^{2}\right)$. Thus, we can define a confidence range $\left(1-Z_{\alpha /2}*\sigma \right)<R_{SVR}<\left(1+Z_{\alpha /2}*\sigma \right)$, where *σ* is the experiential standard variation of $R_{SVR}$ in a static environment, the confidence level is 1-α and the $Z_{\alpha /2}$ value can be gained from *Z-core* table in statistics [23]. Otherwise, the value of $R_{SVR}$ falls below an empirical threshold ($0<R_{SVR}<1-Z_{\alpha /2}*\sigma )$ or exceeds an empirical threshold $R_{SVR}>\left(1+Z_{\alpha /2}*\sigma \right)$. SVR is a lightweight computational scheme that quickly detect abrupt dynamic changes of the environment, as shown in Figure 10.

We note that our window-based variance ratio method differs from the previous methods [5,9]. The previous lightweight human motion detection can be achieve by comparing recent window-based feature measurements to measurements made during a static calibration period when nobody is moving in the area of interest. However, due to mutable indoor scenarios, the static pattern is liable to failure, and re-calibration is frequently executed. On the other hand, the SVR only represents recent signal changes between adjacent windows. When the human is continuously moving, the signal changes between adjacent windows may be similar, and the SVR may fail to detect the human motion in some time, as shown in Figure 10. Thus, to capture the behavior of wireless links when the majority of measurements are likely made while the environment is static and detect continuous signal changes, we apply the coefficient of variation of phase on a long-term time window. We define long-term averaged variance ration (LVR) of phase as:

(13) $$R_{LVR}=\frac{1}{n}\sum_{i=1}^{n}\left|\frac{\delta_{\Delta_{T}}^{i}}{\delta_{\Delta_{LT}}^{i}}\right|$$

where *n* is the number of subcarriers, $\delta_{\Delta_{T}}^{i}$ is the coefficient of variation of phase of $i^{th}$ subcarrier in time intervals $\Delta_{T}$, $\delta_{\Delta_{LT}}^{i}$ is the coefficient of variation of phase of $i^{th}$ subcarrier in a long time intervals $\Delta_{LT}$. $\delta_{\Delta_{LT}}^{i}$ can be obtained from the coefficient of variation of phase of $i^{th}$ subcarrier when the number of corresponding continuous normal SVR is *N*. When no one moves, the $R_{LVR}$ also falls in a confidence interval $\left(1-Z_{\alpha /2}*\lambda \right)<R_{LVR}<\left(1+Z_{\alpha /2}*\lambda \right)$, where *λ* is the experiential standard variation of $R_{LVR}$ in static environment, as shown in Figure 10. The $R_{LVR}$ is calculated based on a stable state when the $R_{SVR}$ is within a normal range for a long time. The $R_{LVR}$ is efficient to identify an abnormal state and a stable state.

The human motion is judged by combining SVR with LVR. We firstly employ the SVR to detect whether an intruder moves into the monitoring area. When the human firstly walks into the area of interest, the value of SVR abruptly changes, as shown in Figure 10. As mentioned above, it is inaccurate to infer whether the intruder further walks into or leaves from the monitoring area. Thus, we can utilize the LVR to track whether the person walks continuously within the monitoring area. If the intruder does not appear in the monitoring area, the $\delta_{\Delta_{LT}}^{i}$ will be update with recent $\delta_{\Delta_{T}}^{i}$.

In the case where there may be multiple antenna pairs, we take the majority vote between antenna pairs over the term window to decide if a human motion event has occurred. More specifically, when a receiver antenna detects a abnormal event, we count the abnormal detections for all the receiver antennas over the $R_{SVR}$ and $R_{LVR}$. For a 2×2 MIMO transmitter and receiver, this would mean computing a majority vote over eight measurements. When the majority of the receiver antennas detect human motion, we infer that a person is moving between the transmitter and the receiver. We will show that this majority vote method improves the performance of our detector by decreasing false alarms and missed detections. We decrease the false alarm rate further by combining temporally close detections together.

## 7. Experiment and Evaluation

The previous sections present the mechanism and design of FRID . In this section, we present the detailed evaluations on the FRID . We interpret the experiment methodology, followed by detailed evaluation results.

### 7.1. Methodology

**Figure 11:**
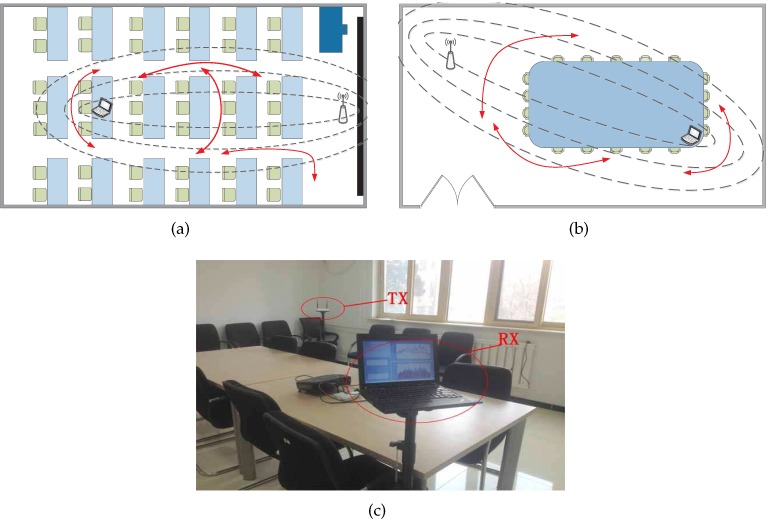
Experimental Scenarios: (**a**) Floor plan in classroom; (**b**) Floor plan in meeting room; (**c**) Experimental equipments.

**Testing Scenarios:** We conduct the measurement campaign in two typical indoor scenarios, as shown in Figure 11, in two academic buildings: A 12.4 m × 7.2 m classroom (Figure 11a) and a meeting room of 5.6 m × 4.2 m (Figure 11b). Each testing room is occupied with desks, chairs, and other furniture, creating different extent of multipath effects.

**Infrastructure Setup:** We employ a single-antenna Tenda W3000R wireless router as the transmitter(TX) operating in IEEE 802.11n AP mode at 2.4 GH. A LENOVO Thinkpad X200 laptop equipped with Intel 5300 NIC and 2 antennas running Ubuntu 10.04 LTS server OS works as the receiver(RX) pinging packets from the transmitter. The firmware is modified as to extract CSIs from data packets using the CSI tools [19]. The transmitter and the receiver are placed about 1.2 m above the floor, as shown in Figure 11c.

**Data Collection:** After setting TX-RX link as in Figure 11, the transmitter is configured to send ICMP packets at the rate of 20 Hz. During the period of online data collection, each sample contains 3000 packets with no movement in the room and 3000 packets with one volunteer moving in the designated line marked by red color, as shown in Figure 11. We recruit 4 volunteers with dynamic speed in total and collect 4 testing samples in the classroom and 3 testing samples in the meeting room.

**Evaluation Metrics:** We mainly focus on the following metrics to evaluate our device-free motion detection system.

- False Negative (FN): The fraction of cases where the receiver fails to detect human motion within the monitoring area.
- False Positive (FP): The fraction of cases where the receiver announces human motions where there is no one.
- Detection Rate (DR): The probability of cases where the receiver makes the right judgement in the existence of human motion.

### 7.2. Performance Evaluation

#### 7.2.1. Overall Performance

**Figure 12:**
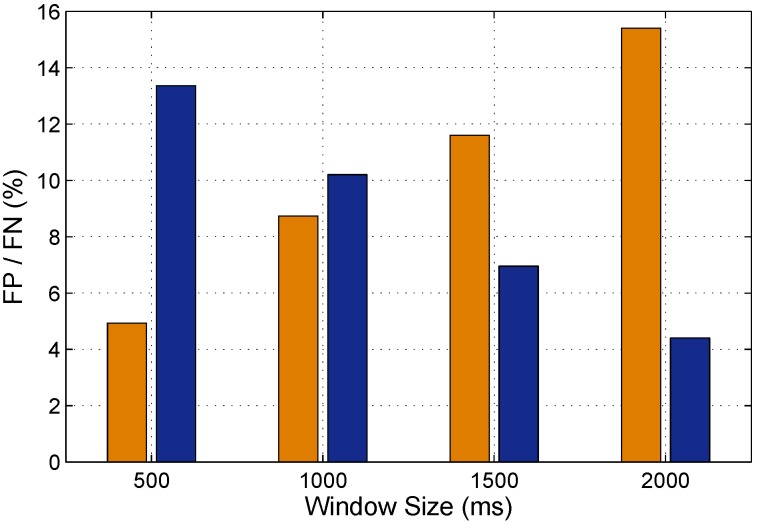
FN and FP of FRID.

**Figure 13:**
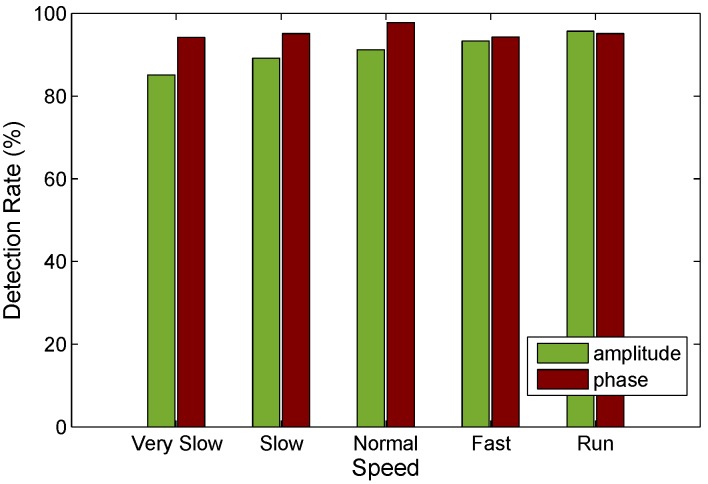
Detection rate of human motions with different movement speed.

**Figure 14:**
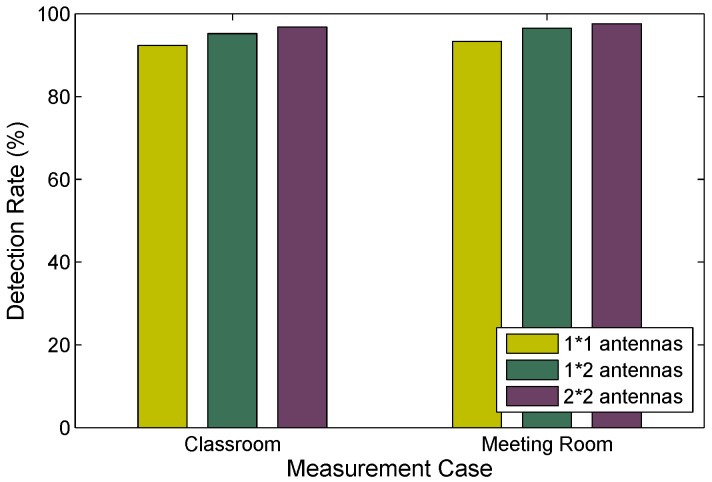
Detection rate of human motions with different number of antennas.

(1) FRID realizes the passive human motion detection based on the variance of phase, but its performance is still satisfactory through our extensive experiments. As depicted in Figure 12, the average FP/FN of FRID is about 10%. In other words, the precision ratio of FRID is above 90%, which is acceptable. Simultaneously, we employ the sliding window for human motion detection, so the size of sliding window play a key role on the performance of detection. Figure 12 illustrates that the FP/FN changes with the sliding window increasing. But the FN and FP is unsynchronized. Choosing a very large window size leads to a high FP rate, but a low FN rate. When the window size is larger, the variance of phase is accordingly larger, which is more sensitive to the human motion but more difficult to distinguish the static scenarios. Thus, the selection of window size provides a tradeoff between false alarm and miss detection.

(2) As mentioned in Section 3, the phase changes are related to human movement distances. The variance of phase in a time window is theoretically different with various motion speeds. To study the performance of FRID under dynamic human motion speeds, we construct numerous experiments in different scenarios when the user walk with dynamic speed. As shown in Figure 13, we can observe that amplitude-based motion detection experiences fall of performance with human walks very slowly, while the performance of FRID remains almost unchanged. Compared with the amplitudes, the phase features are much more sensitive to slow human motion.

(3) In order to study the impacts of majority vote between antenna pairs over the term window on detection performance, we carry out serval testings in the testing scenarios. Figure 14 shows the performance of detection can be improved by using multiple antennas. Due to the environment noise and hardware imperfection, some antenna may be a ”bad” antennas that is not able to accurately detect human motion. The probability that a ”bad” antenna is employed in FRID can be reduced by leveraging multiple antennas [4]. Thus, the majority vote between antenna pairs can effectively preserve the performance of detection rate in FRID.

#### 7.2.2. Comparison with the State-of-the-Art

Furthermore, we pay more attention to the comparison with other representative previous related works: RASID [9] and FIMD [14]. RASID achieves the passive human motion detection based on RSSI. FIMD employs the amplitude of CSIs to realize the passive human motion detection. RASID uses the kernel density estimation technique and relies on the site survey, where normal and abnormal files are collected. FIMD accomplishes the human motion detection combining the eigenvalue of matrix with a clustering technique.

**Figure 15:**
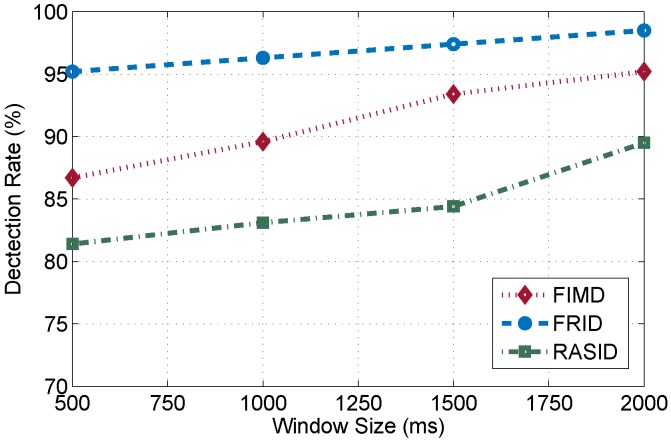
The performance of different DfP systems.

In the section, we contrast the performances of three systems based on a common metric, detection rate. As shown in Figure 15, FIMD performances better than FRID , but RASID is some weaker than FRID . When the human moves on NLOS paths, especially behind the receiver, RSSI fails to distinguish the change of multipath signals. The value of RSSI changes too slightly to detect the human motion. Although FIMD can accurately recognize different environments leveraging the DBSCAN algorithm, it needs to collect too many packets. On the other hand, due to employing amplitudes of CSIs as basic features, the thresholds of FIMD are related to the circumstances and sensitive to the changes of the environment itself. These conditions are challenges to real-time passive human motion detection. By contrast, FRID employs the rate of change of phase, which can eliminate the effect of environment on the thresholds. From the Figure 15, we can observe that when the window size is 2 s (about 40 packets), the detection rate achieves about 95%. FRID can be considered as a light and available real-time passive human motion detection.

#### 7.2.3. Impacts of Confidence Level

**Figure 16:**
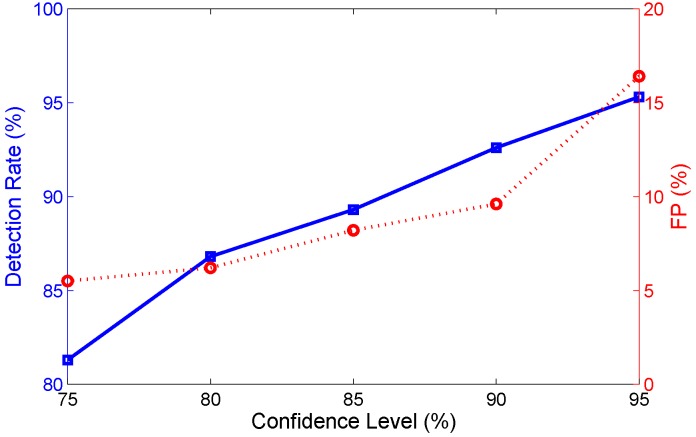
The impacts of confidence level.

As above mentioned in Section 6, the performance of passive human motion detection can be effected by the confidence level (1-α). In the section, we demonstrate the performance of FRID is related with different confidence level. The Figure 16 reveals the inherent tradeoff between the false positive (FP) rate and detection rate. The detection rate and false positive both decrease with the confidence level increasing. When the confidence level increases, the confidence interval becomes narrow. The slight changes of phase caused by the human motion far from the LOS link can be accurately detected but it also proves to cause errors in judgment for the static cases. Hence, in order to improving the detection rate and avoiding the high FP, the confidence coefficient may be set between 80% and 90%.

## 8. Conclusions

In the paper, we propose a novel lightweight and real-time passive human motion detection. With the rapid development of wireless device-free passive localization, indoor fine-grained passive human detection has been widely researched. Real-time passive human detection can be rapidly deployed and needs no massive site survey. Besides, human motion can obviously change the phase of multipath signals. Hence, we achieve a FRID system, a fine-grained real-time passive human motion detection via PHY layer phase information. In order to realize the real-time human motion detection, we develop two schemes: short-term averaged variance ratio (SVR) and long-term averaged variance ratio (LVR). Numerous experiments have proved that the FRID system can achieve high performance, especially for slow human motion. In the future, we will explore to employ more advanced techniques to improve the performance of passive human motion detection via the full CSI information.
